# Quantitative Modeling of Properties in the Extended Critical Region Requires Three-Body Interactions

**DOI:** 10.1021/acs.jctc.6c00794

**Published:** 2026-06-17

**Authors:** Isabel Nitzke, Simon Stephan, Jadran Vrabec

**Affiliations:** † Heat and Mass Transfer, 9376 Otto von Guericke University Magdeburg, 39106 Magdeburg, Germany; ‡ Thermodynamics, Technical University Berlin, 10587 Berlin, Germany

## Abstract

The influence of three-body interactions on thermodynamic response functions in the extended critical region is investigated by using molecular simulations. Results obtained with a high-level *ab initio* two- and three-body potential for krypton are compared to those from the Lennard-Jones potential and a reference equation of state. While the former model shows excellent agreement with the equation of state, the Lennard-Jones potential exhibits significant deviations that cannot be corrected by parameter adjustment, emphasizing the importance of many-body effects for quantitative predictions.

## Graphical abstract

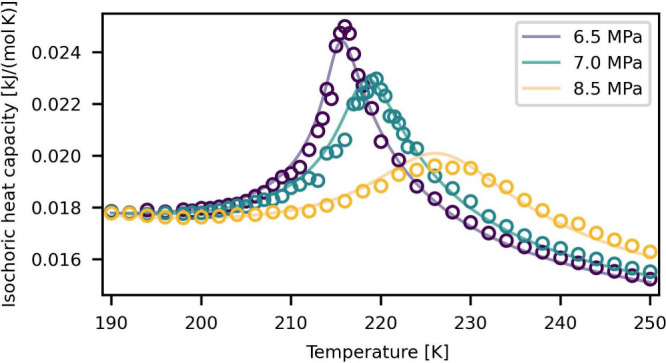

Supercritical fluids exhibit a continuous transition between liquid-like and gas-like behavior in the homogeneous region above the critical point. A concept to mark this transition is the Widom line which also corresponds to the loci of the extrema of thermodynamic response functions.^1^ In recent years, the Widom line has attracted growing attention as a research topic^2–6^ since a thorough understanding of supercritical fluid behavior is crucial for the development and optimization of a wide range of industrial applications and technologies.^7–11^ Despite the widespread and growing use of molecular simulation techniques, a rigorous study of the Widom region remains challenging due to pronounced fluctuations of structural and dynamic properties occurring without a sharp phase separation. Moreover, the thermodynamic response functions near the critical point can exhibit sharp extrema or inflection points. To reliably capture these critical effects, both highly accurate interaction potential models and robust molecular simulation methods are required, as even small deviations can lead to significant errors in the prediction of thermophysical properties.

The vast majority of molecular simulations continue to rely on pairwise interaction potentials because of their computational efficiency and favorable linear scaling with the number of molecules. However, two-body (2B) potentials are inherently limited, as they neglect higher-order interaction terms.^12^ For a long time, this approximation was necessary because of the enormous computational effort required to include many-body interactions. Recently, the increasing availability of high-performance computing resources has made the explicit treatment of three-body (3B) interactions more feasible. Nonetheless, 3B interaction potentials typically involve a large number of parameters that must be determined through fitting procedures. In addition, the growing emphasis on the responsible use of computational resources requires careful consideration of their application, particularly because of the significantly higher computational cost compared with pairwise models. In this work, the influence of 3B interactions on thermodynamic response functions in the Widom region was studied and compared to the classical 2B approach.

The Lennard-Jones (LJ) potential^13,14^

1 $$u_{\mathrm{LJ}}\left(r_{ij}\right)=4\varepsilon \left[{\left(\frac{\sigma }{r_{ij}}\right)}^{12}-{\left(\frac{\sigma }{r_{ij}}\right)}^{6}\right]$$

is one of the most commonly used pair potentials and was therefore employed in this work. As shown by Rutkai et al.,^15^ the LJ potential with appropriate parameters for size σ and energy *ε* provides a suitable representation of simple fluids like the noble gases. Accordingly, krypton was investigated here. When 3B interactions are explicitly considered, the total interaction energy is decomposed into a 2B and a nonadditive 3B contribution

2 $$U=\underset{i<j}{\sum }u_{2B}\left(r_{ij}\right)+\underset{i<j<k}{\sum }\Delta u_{3B}\left(r_{ij},r_{jk},r_{ik}\right)$$

Following the krypton model proposed by Jäger et al.,^16^ both contributions have been implemented into the simulation software *ms*2.^17,18^ The 2B interactions are described by an augmented Tang–Toennies (TT) potential

3 $$u_{2B}\left(r_{ij}\right)=A\exp\left(a_{1}r_{ij}+a_{2}r_{ij}^{2}+a_{-1}r_{ij}^{-1}\right)-\overset{8}{\underset{n=3}{\sum }}f_{2n}\left(r_{ij}\right)\frac{C_{2n}}{r_{ij}^{2n}}$$

with *f*_2*n*_ denoting damping functions of the form

4 $$f_{2n}\left(r_{ij}\right)=1-\exp\left(-br_{ij}\right)\overset{2n}{\underset{k=0}{\sum }}\frac{{\left(br_{ij}\right)}^{k}}{k!}$$

The nonadditive 3B interactions are described by an extended Axilrod–Teller–Muto potential

5 $$\Delta u_{3B}\left(r_{ij},r_{jk},r_{ik}\right)=\left(1+3\cos\vartheta_{ij}\cos\vartheta_{ik}\cos\vartheta_{jk}\right)\times \left(\frac{C_{ATM}}{{\left(r_{ij}r_{ik}r_{jk}\right)}^{3}}+\exp\left(-\alpha \left(r_{ij}+r_{ik}+r_{jk}\right)\right)\overset{5}{\underset{n=0}{\sum }}A_{2n}{\left(r_{ij}r_{ik}r_{jk}\right)}^{2n/3}\right)$$

All parameters were taken from ref.^16^ For the 2B potential, these include *A*, *a*_1_, *a*_2_, *a*_–1_, *b*, and *C*_2*n*_ (*n* = 3, ..., 8), while the 3B potential was parametrized by *C*_*ATM*_, α, and *A*_2*n*_ (*n* = 0, ..., 5).

The TT potential^19^ was developed to provide a physically motivated description of intermolecular interactions by combining a short-range repulsive term with a damped dispersion expansion. As shown in previous work,^12^ its increased flexibility leads to limited improvements over the LJ potential for most thermodynamic properties, while significant deviations from reference data persist. Consequently, the availability of the high-accuracy krypton potential^16^ offers the opportunity to investigate whether the remaining shortcomings can be attributed to the absence of many-body interactions.

The behavior of both models can be analyzed with reference to the equations of state (EOS) by Thol et al.,^20^ as well as Lemmon and Span,^21^ which rest on fundamentally different data. The EOS by Thol et al.^20^ was developed to encode the LJ model fluid and is therefore expected to reproduce LJ simulation results consistently. Krypton behavior is only obtained through the parametrization of σ and *ε* suggested by Rutkai et al.^15^ In contrast, the EOS by Lemmon and Span^21^ describes real krypton and was fitted to high-accuracy experimental data. It thus provides a reliable reference for assessing both interaction models and determines the critical point at *T*_*c*_ = 209.5 K and *p*_*c*_ = 5.526 MPa. For the LJ model, the reduced critical properties *T*_*c*_ *k*_B_/*ε* = 1.32 and *p*_*c*_σ^3^/*ε* = 0.13 correspond to *T*_*c*_ = 214.6 K and *p*_*c*_ = 6.114 MPa when using the parameters of Rutkai et al.^15^ In a previous study,^12^ the limitations of the LJ and TT potentials have been investigated over a wide range of state points across the fluid region. These limitations are not specific to the extended critical region but arise more generally in dense fluids. The present work focuses on the extended critical region, which represents a particularly challenging regime where many-body effects are expected to be especially pronounced. For the comparison of the LJ and 2B+3B potential models, a temperature range from 190 to 250 K was covered along the isobars of 6.5, 7, and 8.5 MPa, as illustrated in Figure 1.

**Figure 1:**
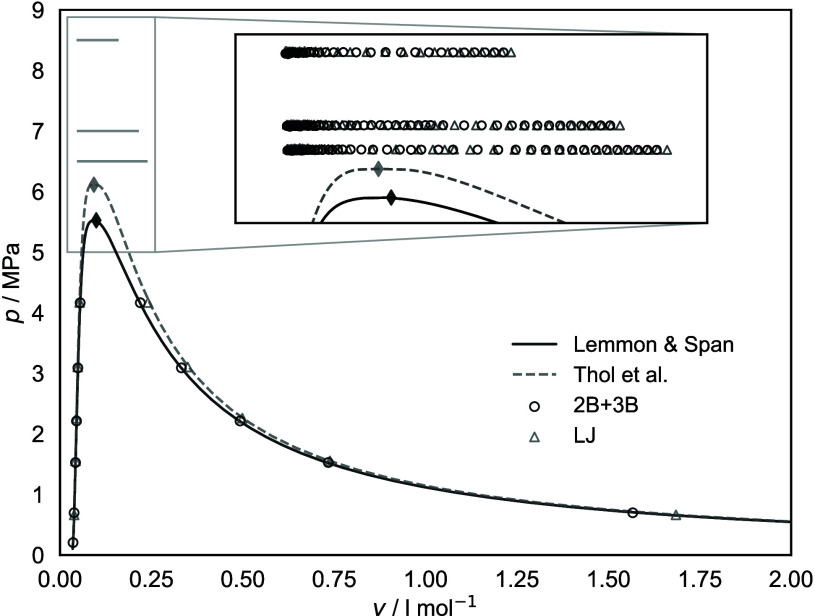
Representation of simulated states using LJ and 2B+3B potential models relative to the vapor–liquid equilibria from the EOS by Thol et al.,^20^ as well as Lemmon and Span.^21^

While accurate interaction potentials are essential, the reliable determination of thermodynamic response functions also depends on the employed simulation methodology. Although techniques for the sampling of different statistical ensembles have long been available, thermodynamic response functions were largely inaccessible at first. A profound formalism suggested by Lustig provided the entire set of according expressions for the microcanonical (*NVE*) and canonical (*NVT*) ensembles.^22–27^ This formalism was only recently extended to the remaining statistical ensembles in the meticulous works of Ströker et al.^28–32^ The resulting expressions were fully integrated into the latest version of the molecular simulation software *ms*2^18^, making it a powerful tool for the rigorous analysis of phenomena in the extended critical region. In particular, both molecular dynamics (MD) and Monte Carlo (MC) simulations provide the isochoric and isobaric heat capacities *c*_*v*_ and *c*_*p*_, the thermal expansion coefficient α_*p*_, the isothermal compressibility β_*T*_, the speed of sound *w*, and the Joule-Thomson coefficient μ_*JT*_ in any ensemble.

As shown in previous work,^33^ thermodynamic property data obtained from simulations in different ensembles are generally consistent. However, in the extended critical region, enhanced fluctuations can lead to an increased ensemble dependence of the second-order thermodynamic response functions. Moreover, in isochoric ensembles, the explicit calculation of the virial is required, which significantly increases the computational cost, particularly when 3B interactions are considered. To investigate this influence, MC simulations were first performed using the computationally efficient LJ potential in all ensembles. Simulation details and the resulting data are provided in the Supporting Information. As expected,^33^ results obtained in all eight ensembles agree within statistical uncertainties outside the critical region, with the locations of the extrema being qualitatively consistent. Nevertheless, clear differences are observed regarding the magnitudes of the statistical uncertainties. In particular, isochoric ensembles exhibit strong fluctuations such that the results lose quantitative reliability. In contrast, the closed isobaric–isothermal (*NpT*) ensemble yields the most stable results and was therefore found to be most suited for analyzing second-order thermodynamic properties in the extended critical region.

Based on these findings, additional simulations were performed in the *NpT* ensemble with the 2B+3B potential. Figure 2 summarizes the isobaric and isochoric heat capacities *c*_*v*_ and *c*_*p*_, thermal expansion coefficient α_*p*_, and isothermal compressibility β_*T*_. At the considered pressures, all properties exhibit the expected nonlinear behavior with pronounced maxima, which remain clearly visible even on a logarithmic scale and are characteristic of the extended critical region.

**Figure 2:**
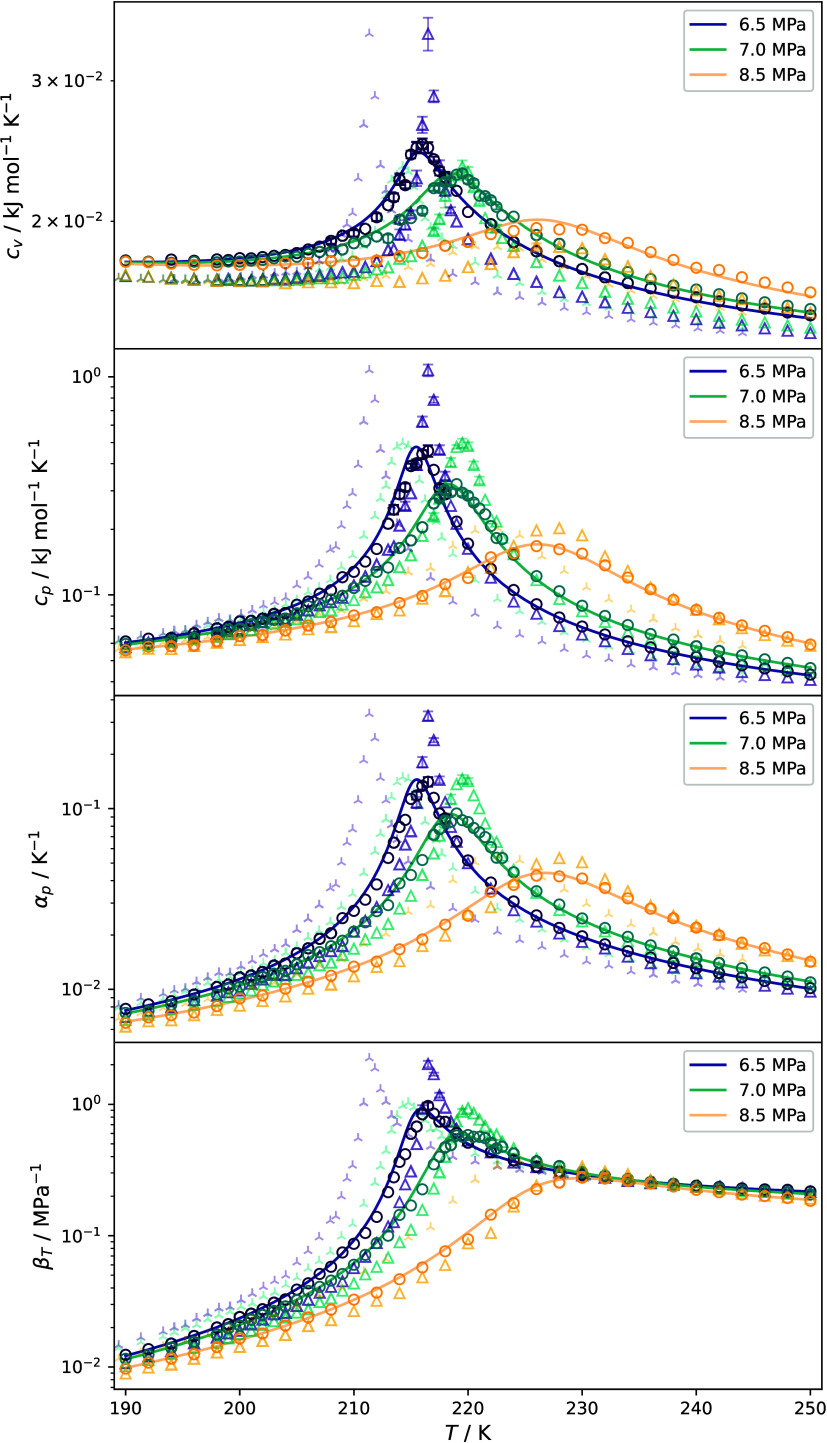
Temperature dependence of thermodynamic response functions of krypton along isobars. Simulation results using the LJ and 2B+3B potential are represented by triangles and circles, respectively. Solid lines represent the EOS by Lemmon and Span.^21^ Statistical uncertainties within symbol size were omitted. The triangular tick markers correspond to a LJ potential which was modified such that its critical point coincides with that of the EOS by Lemmon and Span.

Moreover, simulation results for the 2B+3B potential are in excellent agreement with the EOS by Lemmon and Span.^21^ This Helmholtz energy EOS employs a functional form with 12 coefficients that were fitted to high-quality experimental data. It provides a reliable descriptive performance and remains robust even in regions that are typically challenging for empirical EOS. Notably, its functional form was not developed specifically for krypton, but rather adopted from a class-based optimization approach. The molecular simulations, in contrast, are based on a high-accuracy krypton interaction potential exclusively derived from quantum-chemical *ab initio* calculations by Jäger et al.^16^ The corresponding potential energy curve was obtained from interaction energies evaluated at several intermolecular separations using coupled-cluster methods up to perturbative triple excitations, with additional contributions from higher-order terms. Further refinements include extrapolation to the complete basis set limit, as well as the inclusion of core-valence correlation and relativistic effects. The consistency between both approaches therefore highlights not only the accuracy of the compact, transferable EOS,^21^ but also the reliability of the state-of-the-art *ab initio* interaction potential.^16^ This can also be observed in Figure 3 for the density ρ, speed of sound *w*, and Joule–Thomson coefficient μ_*JT*_.

**Figure 3:**
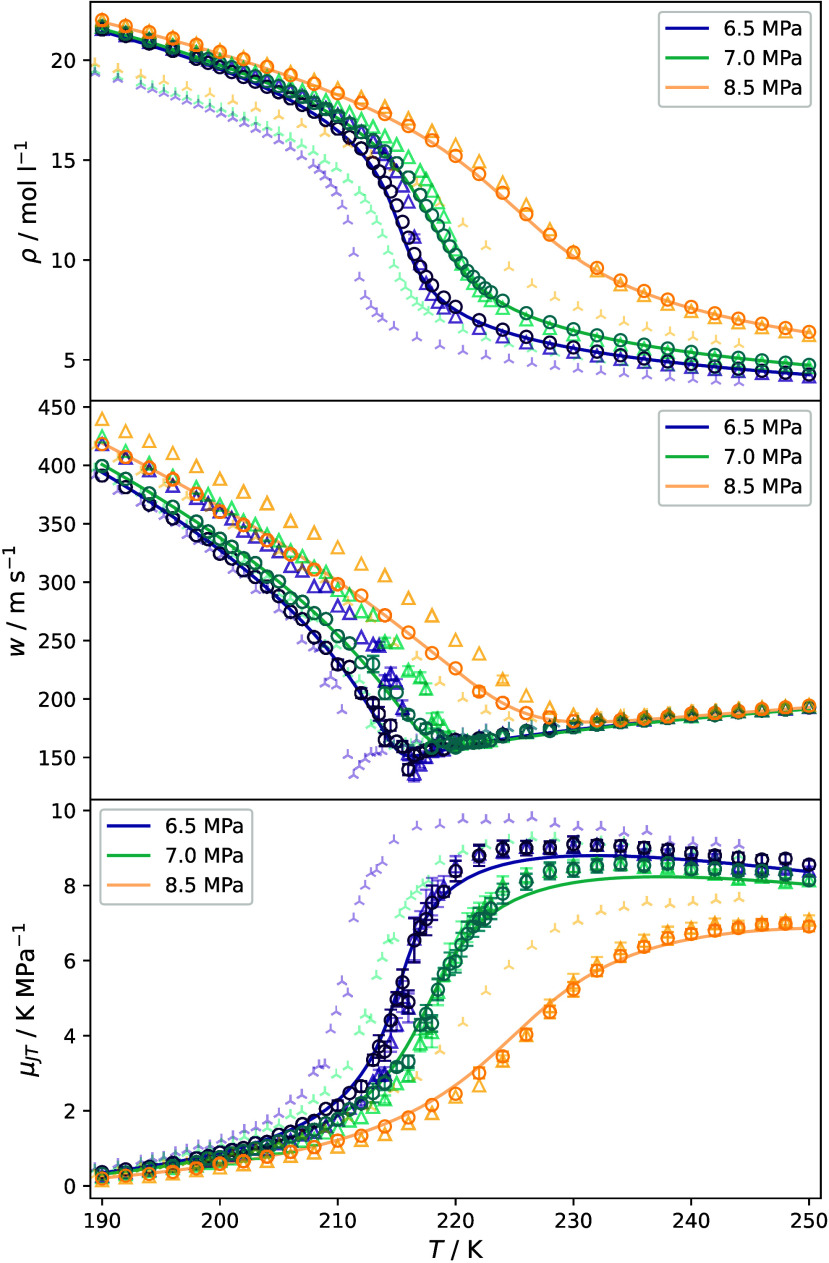
Temperature dependence of density and thermodynamic response functions of krypton along isobars. Simulation results using the LJ and 2B+3B potential are represented by triangles and circles, respectively. Solid lines represent the EOS by Lemmon and Span.^21^ Statistical uncertainties within symbol size were omitted. The triangular tick markers correspond to a LJ potential which was modified such that its critical point coincides with that of the EOS by Lemmon and Span.

Regarding the LJ potential, the maxima in Figure 2 are significantly more pronounced. This can be attributed to the model-dependent difference in the location of the critical point: The lowest isobar at 6.5 MPa corresponds to a pressure of *p* = 1.18*p*_*c*_ for the 2B+3B potential and *p* = 1.06*p*_*c*_ for the LJ potential, leading to a more pronounced manifestation of critical effects. To further assess the limitations of the LJ model, another parametrization was considered in addition to the original parameters for krypton by Rutkai et al.^15^ To this end, the LJ parameters were adjusted to exactly reproduce the critical point according to the EOS by Lemmon and Span,^21^ yielding σ = 3.7223 Å and *ε*/*k*_B_ = 158.712 K. Owing to the scaling properties of the LJ potential, simulation results obtained with the original parametrization can be rescaled accordingly to the modified one. These modified data are also depicted in Figures 2 and 3. Enforcing agreement at the critical point might be expected to improve consistency with the reference EOS. However, the opposite was observed: the rescaled results further increase the deviations. This demonstrates that the limitations of the LJ model in the extended critical region cannot be resolved by parameter adjustment alone but are inherent to its pairwise nature. Therefore, qualitatively and quantitatively accurate predictions require the explicit consideration of many-body interactions.

Thermodynamic response functions up to second order in the extended critical region were investigated by using molecular simulations. MC simulations in eight statistical ensembles yielded qualitatively consistent results. However, the *NpT* ensemble proved to be most suitable for further analyses due to its comparatively small statistical uncertainties and robustness. The LJ model fails to reproduce the thermodynamic properties quantitatively. Although parameter adjustment can shift the critical point to match the reference, it further increases the deviations from the reference EOS. In contrast, simulations employing an *ab initio* 2B+3B potential show excellent agreement with the EOS. These results underline the importance of many-body contributions for studies in the extended critical region, when truly quantitative data are needed.

## Supplementary Material



## Notes

Simulation results for second-order response function of the LJ potential in eight statistical ensembles (PDF)
