# Supplementary material for: Quantitative Modeling of Properties in the Extended Critical Region Requires Three-Body Interactions
Source: J Chem Theory Comput. 2026 Jun 17;22(13):6290–4. doi: 10.1021/acs.jctc.6c00794 (PMC13374038; doi:10.1021/acs.jctc.6c00794)
Supplement: Supplementary file 1 [file ct6c00794_si_001.pdf]

Supporting Information to: Quantitative Modeling of Properties in the Extended Critical Region Requires Three-Body Interactions

Isabel Nitzke[1], Simon Stephan[1], and Jadran Vrabec*[2]

[1]Heat and Mass Transfer, Otto von Guericke University Magdeburg, 39106 Magdeburg, Germany

[2]Thermodynamics, Technical University Berlin, 10587 Berlin, Germany

*Email: vrabec@tu-berlin.de

Monte Carlo simulations with the Lennard-Jones potential using interaction parameters for Krypton from Rutkai et al.[1] were performed in eight statistical ensembles. At pressures of 6.5, 7, and 8.5 MPa, a temperature range from 190 K to 250 K was covered. All individual state points were initially equilibrated with 2048 molecules for 10^4 cycles in the canonical ensemble, followed by 2×10^4 cycles in the respective ensemble used for the production run which entailed 8×10^5 cycles. The cutoff radius was set to 17.5 Å. Statistical uncertainties were estimated with a block averaging method proposed by Flyvbjerg and Petersen[2] and the law of error propagation.

References

[1] Rutkai, G.; Thol, M.; Span, R.; Vrabec, J. How well does the Lennard-Jones potential represent the thermodynamic properties of noble gases? *Mol. Phys.* **2016**, *115*, 1104–1121.

[2] Flyvbjerg, H.; Petersen, H. G. Error estimates on averages of correlated data. *J. Chem. Phys.* **1989**, *91*, 461–466.

[3] Lemmon, E. W.; Span, R. Short Fundamental Equations of State for 20 Industrial Fluids. *J. Chem. Eng. Data* **2006**, *51*, 785–850.

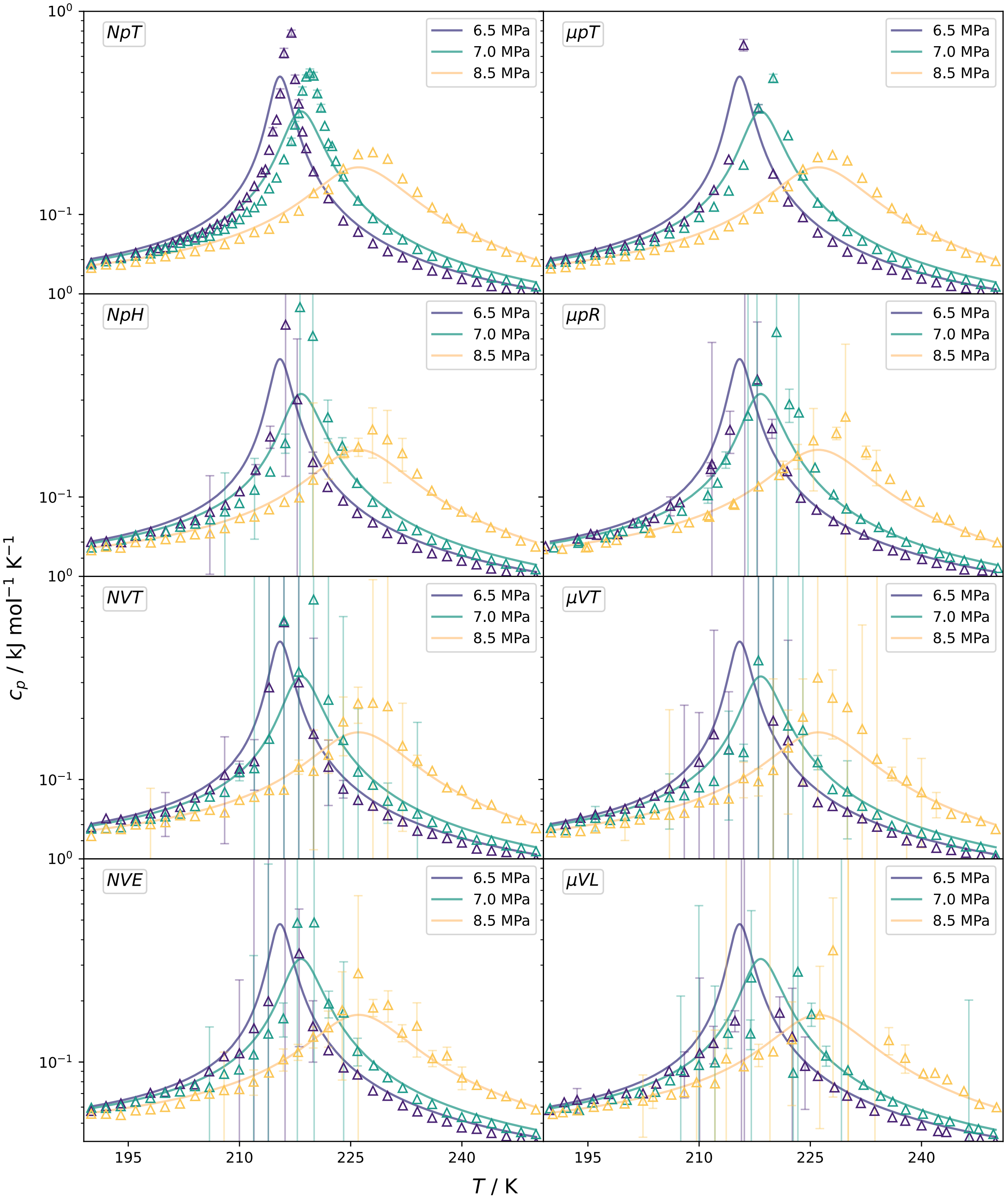

Figure S1: Simulation results for the isobaric heat capacity sampled with the LJ potential (triangles) in all eight ensembles compared to the EOS of Lemmon and Span (solid lines).[3]

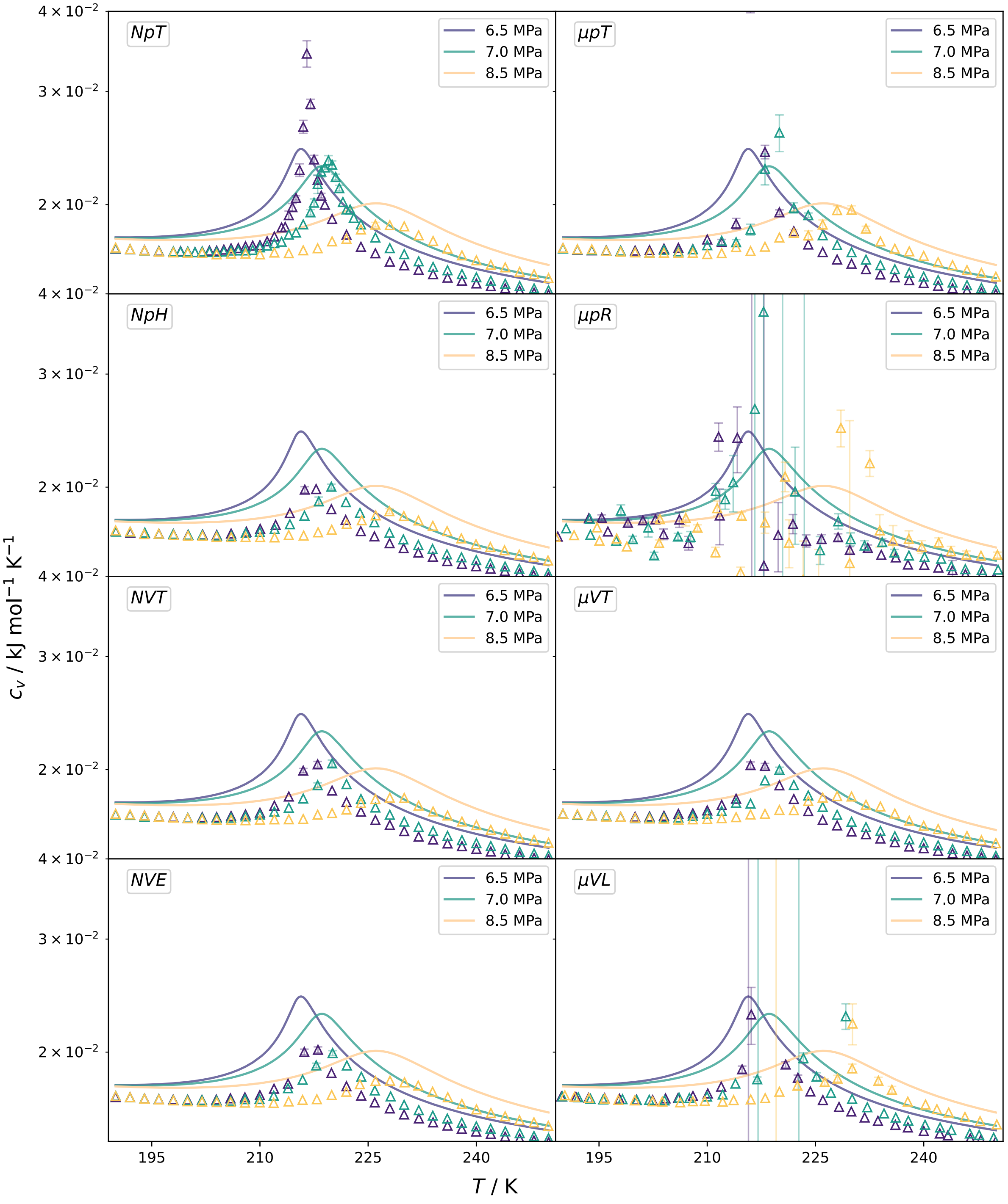

Figure S2: Simulation results for the isochoric heat capacity sampled with the LJ potential (triangles) in all eight ensembles compared to the EOS of Lemmon and Span (solid lines).[3]

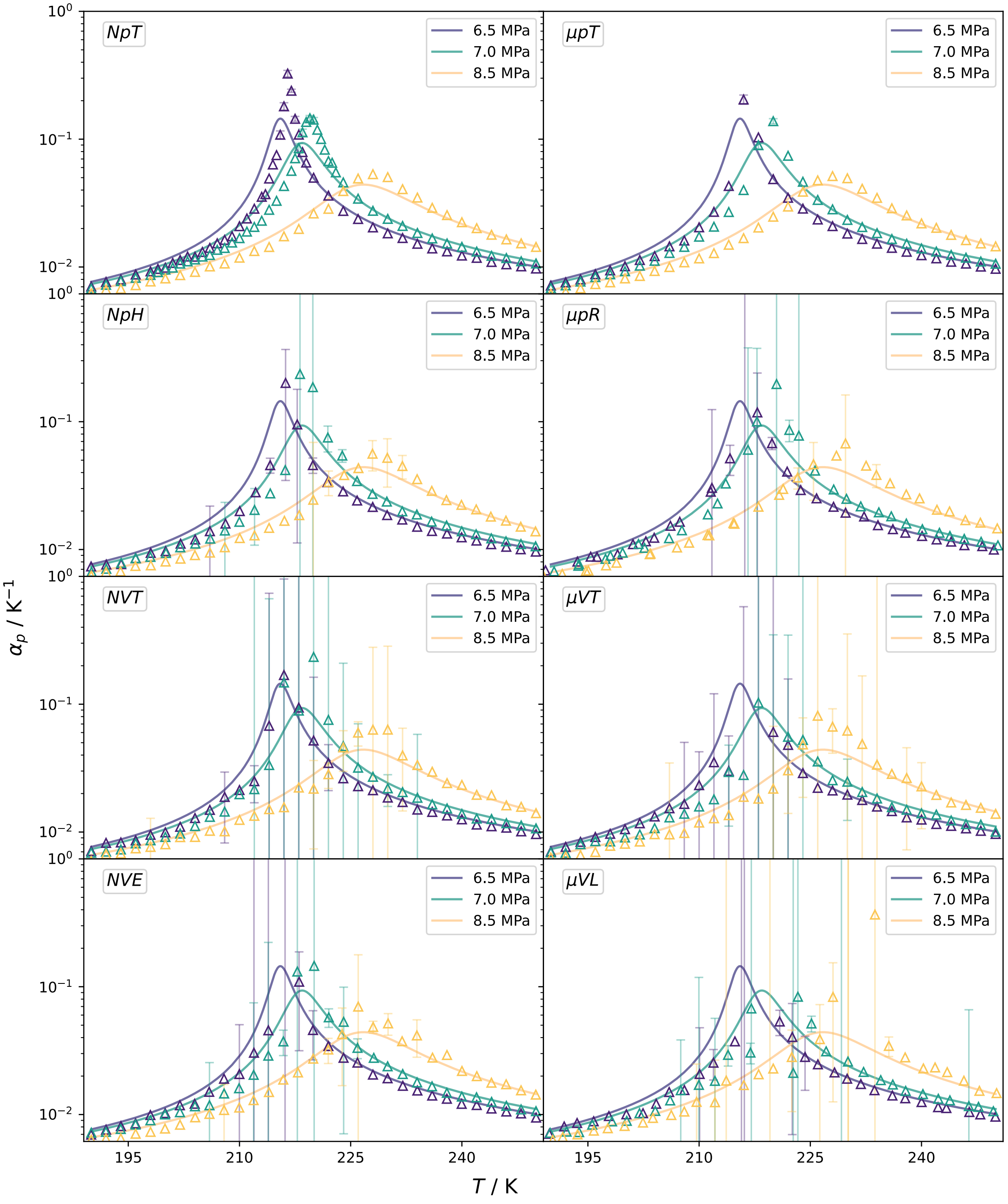

Figure S3: Simulation results for the thermal expansion coefficient sampled with the LJ potential (triangles) in all eight ensembles compared to the EOS of Lemmon and Span (solid lines).[3]

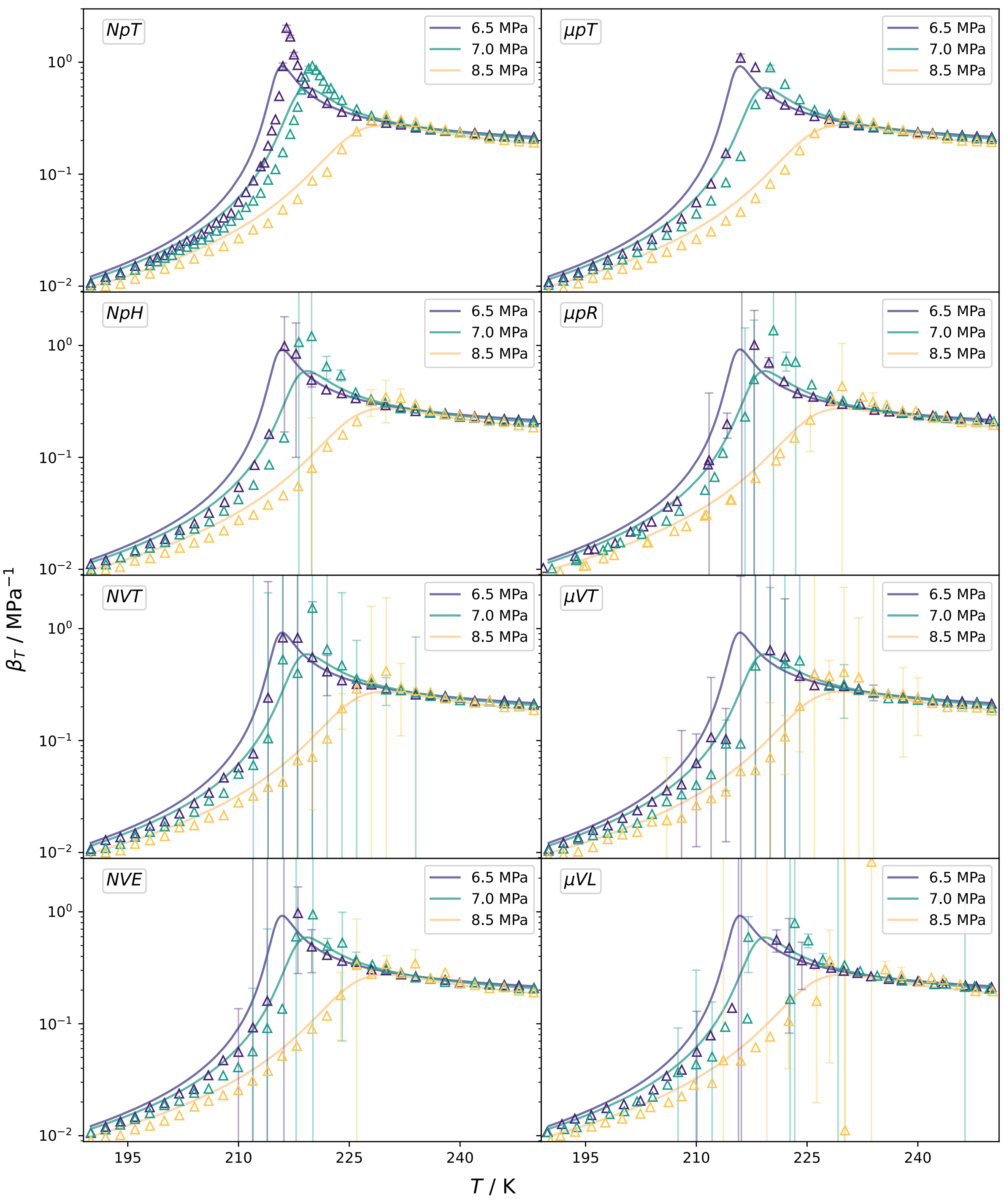

Figure S4: Simulation results for the isothermal compressibility sampled with the LJ potential (triangles) in all eight ensembles compared to the EOS of Lemmon and Span (solid lines).[3]

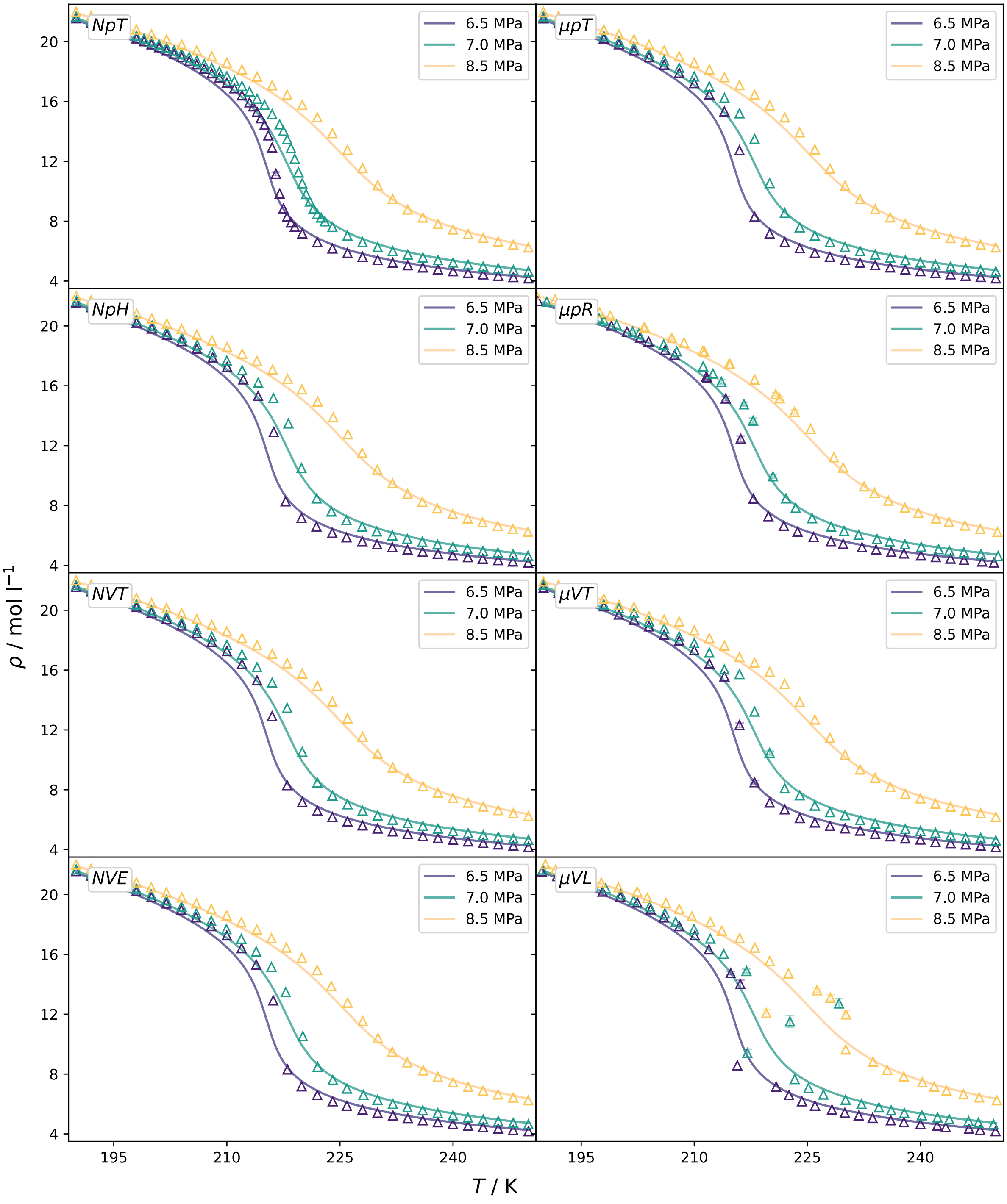

Figure S5: Simulation results for the density sampled with the LJ potential (triangles) in all eight ensembles compared to the EOS of Lemmon and Span (solid lines).[3]

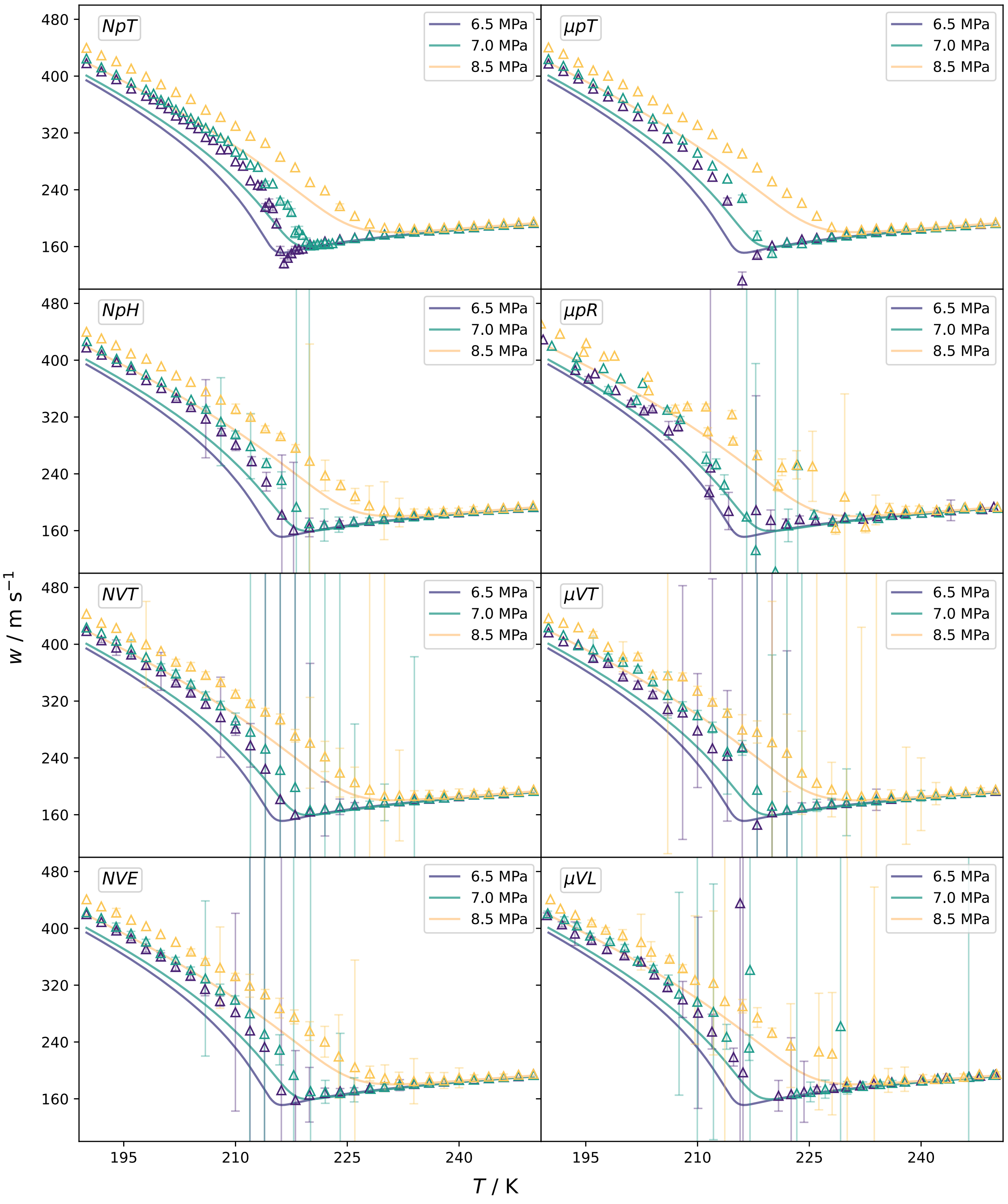

Figure S6: Simulation results for the speed of sound sampled with the LJ potential (triangles) in all eight ensembles compared to the EOS of Lemmon and Span (solid lines).[3]

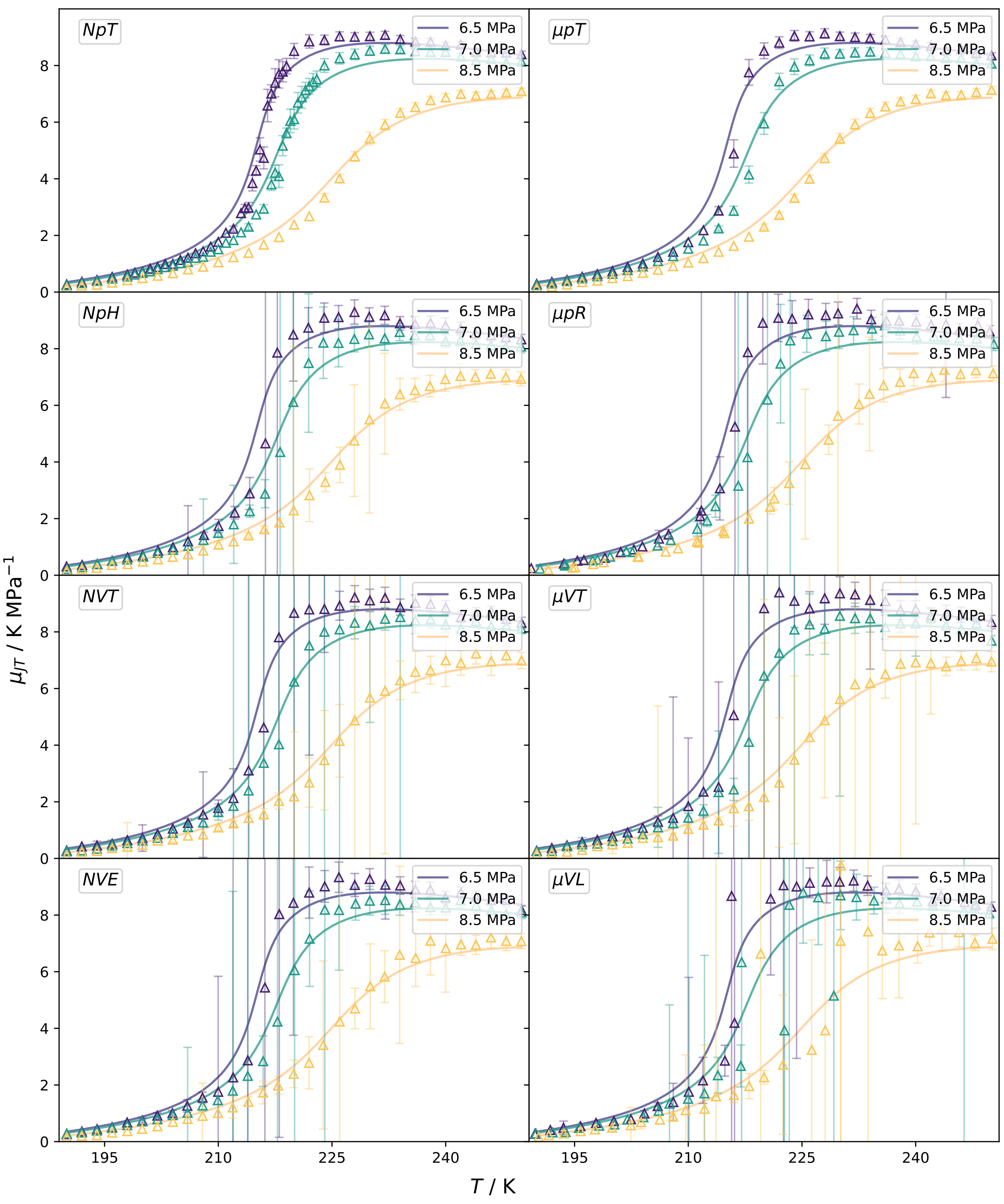

Figure S7: Simulation results for the Joule-Thomson coefficient sampled with the LJ potential (triangles) in all eight ensembles compared to the EOS of Lemmon and Span (solid lines).[3]
